# *Agrobacterium tumefaciens* ferritins play an important role in full virulence through regulating iron homeostasis and oxidative stress survival

**DOI:** 10.1111/mpp.12969

**Published:** 2020-07-17

**Authors:** Jing Yang, Xiaoyue Pan, Yujuan Xu, Yuan Li, Nan Xu, Zhiwei Huang, Jingyang Ye, Dawei Gao, Minliang Guo

**Affiliations:** ^1^ College of Bioscience and Biotechnology Yangzhou University Jiangsu Province Yangzhou City China

**Keywords:** *Agrobacterium tumefaciens*, bacterioferritin, Dps ferritin, iron homeostasis, oxidative stress resistance, pathogenicity, tumorigenesis

## Abstract

Ferritins are a large family of iron storage proteins, which are used by bacteria and other organisms to avoid iron toxicity and as a safe iron source in the cytosol. *Agrobacterium tumefaciens*, a phytopathogen, has two ferritin‐encoding genes: *atu2771* and *atu2477*. *Atu2771* is annotated as a Bfr‐encoding gene (Bacterioferritin, Bfr) and *atu2477* as a Dps‐encoding gene (DNA binding protein from starved cells, Dps). Three deletion mutants (Δ*bfr*, Δ*dps*, and *bfr*‐*dps* double‐deletion mutant ΔbdF) of these two ferritin‐encoding genes were constructed to investigate the effects of ferritin deficiency on the iron homeostasis, oxidative stress resistance, and pathogenicity of *A. tumefaciens*. Deficiency of two ferritins affects the growth of *A. tumefaciens* under iron starvation and excess. When supplied with moderate iron, the growth of *A. tumefaciens* is not affected by the deficiency of ferritin. Deficiency of ferritin significantly reduces iron accumulation in the cells of *A. tumefaciens*, but the effect of Bfr deficiency on iron accumulation is severer than Dps deficiency and the double mutant ΔbdF has the least intracellular iron content. All three ferritin‐deficient mutants showed a decreased tolerance to 3 mM H_2_O_2_ in comparison with the wild type. The tumour induced by each of three ferritin‐deficient mutants is less than that of the wild type. Complementation reversed the effects of ferritin deficiency on the growth, iron homeostasis, oxidative stress resistance, and tumorigenicity of *A. tumefaciens*. Therefore, ferritin plays an important role in the pathogenesis of *A. tumefaciens* through regulating iron homeostasis and oxidative stress survival.

## Graphical abstract

Deficiency of two *Agrobacterium tumefaciens* ferritins, Bfr and Dps, not only affects the growth under iron stress, reduces the iron accumulation, and impairs the oxidative resistance, but also attenuates the virulence.

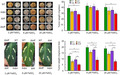

## 1 INTRODUCTION

*Agrobacterium tumefaciens* is a ubiquitous soilborne gram‐negative bacterium that has two lifestyles: independent free‐living or acting as a plant pathogen that leads to the crown gall tumour disease in more than 140 species of dicotyledonous plants (Guo *et al*., 2011; Pacurar *et al*., 2011). In the last century, the crown gall tumour disease once caused significant yield loss in many perennial horticultural crops (e.g., apple, cherry, and grape) and was considered as the major problem of horticultural production (Kado, 2014). *A. tumefaciens* causes the neoplastic growth of the infected cells by transferring a DNA fragment (called the transferred DNA, or T‐DNA) from its Ti plasmid to the host cell genome and genetically transforming the host cell (Pacurar *et al*., 2011). The ability of *A. tumefaciens* to genetically transform a wide variety of plant species has allowed it to evolve from a phytopathogen to the most powerful genetic transformation tool (Guo *et al*., 2019; Huang *et al*., 2019). Because the wild‐type coding region in the T‐DNA can be replaced by any DNA fragment without any effect on the T‐DNA trafficking from *A. tumefaciens* to a host cell, *Agrobacterium*‐mediated genetic transformation has become the most popular technique to deliver genetic material into plants (Yang *et al*., 2015; Guo *et al*., 2017). Therefore, interest in the study of *A. tumefaciens* has changed from controlling crown gall tumour disease to understanding the molecular mechanism of how *A. tumefaciens* transfers its T‐DNA to the host cell (Matveeva and Lutova, 2014; Singh and Prasad, 2016).

Current research interest in *A. tumefaciens* is mainly focused on how to increase the T‐DNA transfer efficiency and broaden the host range (Guo *et al*., 2019). Iron restriction is often the critical environmental stress for plant pathogens to infect plant hosts (Sagova‐Mareckova *et al*., 2015; Pandey *et al*., 2016). To increase the infection efficiency of *A. tumefaciens* to the host, both the minimum AB medium and induction broth (IB medium) are supplied with plenty of iron for culturing *A. tumefaciens* under laboratory conditions (Heindl *et al*., 2015). Ferritins are common in all three domains of life and function as the cellular repository of excess iron to achieve iron homeostasis (He and Marles‐Wright, 2015). Besides iron metabolism, ferritins were reported to influence other biological processes, such as resistance to stressful conditions (Arosio *et al*., 2009; Oliveira *et al*., 2020), biofilm formation (Heindl *et al*., 2015; Soldano *et al*., 2020), expression of some genes (Wei *et al*., 2012; Pandey *et al*., 2016; Sankari and O’Brian, 2016; Oliveira *et al*., 2018), bacterial growth (Abdul‐Tehrani *et al*., 1999), and pathogenicity of pathogens (Boughammoura *et al*., 2008; Sharma and Bisht, 2017). Therefore, ferritins may play important roles in various biological processes of *A. tumefaciens*. In this report, we aim to elucidate the effects of ferritins on the pathogenicity of *A. tumefaciens*.

Ferritins are ubiquitous ancient proteins that are also iron‐storage proteins because of their indispensable function in maintaining iron homeostasis (Bradley *et al*., 2016). When the intracellular soluble iron (Fe^2+^) is excessive, ferritins are able to mineralize and store the iron, and thus protect the cells from the damage caused by iron‐induced reactive hydroxyl radicals, which are produced by the Fenton reaction (Fe^2+^ + H_2_O_2_ → Fe^3+^ + •OH + OH^–^). Under iron‐starvation conditions, the insoluble form Fe(III) in the ferritins can be reduced to the soluble form Fe(II) and released for metabolism (Cornelis *et al*., 2011; Gozzelino and Arosio, 2016). Ferritins are part of the superfamily of ferritin‐like proteins that share a four α‐helix bundle structure domain and possess iron‐storage capacity.

In bacteria, there are at least three types of ferritin‐like proteins: the archetypal ferritins (Ftn), the haem‐containing bacterioferritins (Bfr), and the DNA‐binding protein from starved cells (Dps) (Andrews, 2010). Bacterial Ftn proteins are similar to those found in eukaryotes. Bfr proteins are found only in bacteria and archaea (Weeratunga *et al*., 2009; Chen *et al*., 2010). Both Ftn and Bfr proteins possess similar structure and the same function, iron storage. The Dps proteins are smaller than Ftn and Bfr proteins and are found only in prokaryotes (Honarmand Ebrahimi *et al*., 2015). Dps proteins usually bind DNA nonspecifically to protect it from harmful hydroxyl radicals produced by the Fenton reaction (Andrews, 2010; Theil *et al*., 2013). In many cases, all three ferritin‐like proteins can coexist in the same bacterium (Bai *et al*., 2015; Sharma and Bisht, 2017).

Unlike many bacteria, the genome of *A. tumefaciens* C58 carries only two genes that are predicted to encode the ferritin‐like proteins. The two ferritin‐encoding genes are numbered as *atu2771* and *atu2477*, which are respectively annotated as *bfr* and *dps* genes. It is unclear why *A. tumefaciens* lacks the ubiquitous archetypal ferritin Ftn. The uncommon ferritin combination of *A. tumefaciens* may lead to a unique function of Bfr and Dps in this bacterium. Additionally, it was reported that ferritin deficiency could significantly attenuate the pathogenicity of many pathogens (Waidner *et al*., 2002; Velayudhan *et al*., 2007; Boughammoura *et al*., 2008). Because the main function of ferritin is to store iron and plenty of iron should be supplied for growing and inducing *A. tumefaciens* cells, here we will explore the physiological function of ferritin in *A. tumefaciens* with the emphasis on the roles of ferritin in the tumorigenesis of *A. tumefaciens*.

## 2 RESULTS

### 2.1 *A. tumefaciens* genome has only two ferritin‐encoding genes

**Figure 1:**
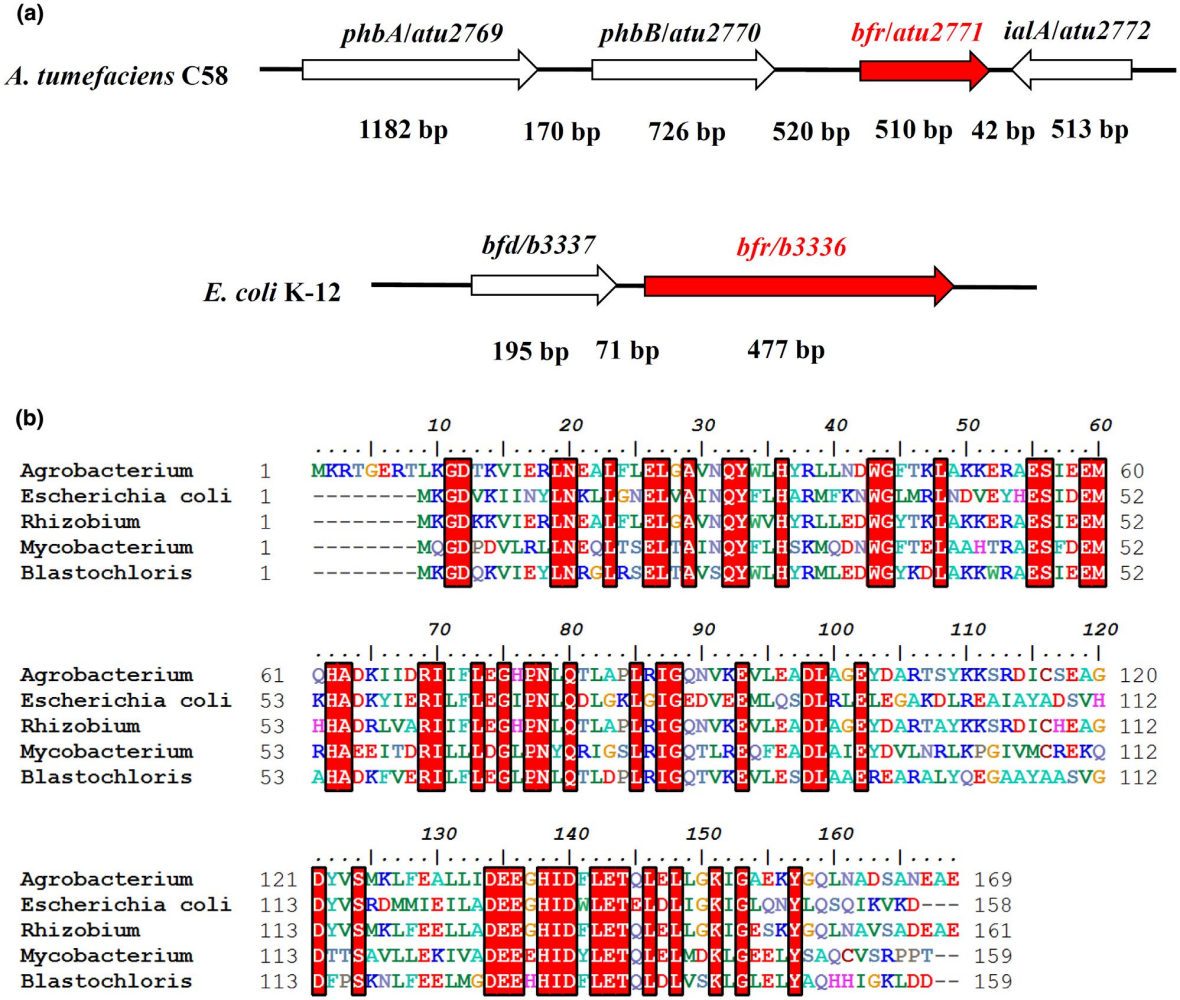
The gene organization adjacent to *bfr* and the Bfr protein sequence alignment. (a) The adjacent gene organization of the *Agrobacterium tumefaciens* *bfr* gene is different from that of the typical bacterium, *Escherichia coli*, in which a *bfd* gene coexists in the same operon as the *bfr* gene. The annotated gene name is labelled above the box arrow and the length of the gene fragment (or the spacer between genes) is labelled below the box arrow. The products encoded by the genes of *phbA*, *phbB*, *bfr*, *ialA*, and *bfd* are annotated as acetyl‐CoA acetyltransferase, acetoacetyl CoA reductase, bacterioferritin, invasion protein A, and bacterioferritin‐associated ferredoxin, respectively. (b) Sequence alignment of Bfr proteins from five different bacteria shows the similarity of the amino acid and the haem‐binding methionine (M60). The numbers indicate the amino acid positions

Ferritin genes are found throughout the biological world. Prokaryotes show considerable numbers of ferritin genes (Bai *et al*., 2015). Some bacteria contain all three types of ferritin: Ftn, Bfr, and Dps (Rivera, 2017; Sharma and Bisht, 2017), but *A. tumefaciens* only has two types of ferritin and one copy of the gene for each ferritin. The gene *atu2771* is annotated as *bfr* and *atu2477* is annotated as *dps*. Bfr protein is very similar to Ftn protein in both structure and function. Both Ftn and Bfr from bacteria are composed of 24 identical subunits and each subunit invariably contains a ferroxidase catalytic centre. The main difference between bacterial Ftn and Bfr is the presence of haem in the Bfr (Arosio *et al*., 2009). Ferritin genes are highly conserved and play an essential role in all living organisms. In some bacterial genomes, the Ftn‐encoding gene (*ftn*) was often misannotated as the *bfr* gene (Yao *et al*., 2011; Rivera, 2017). Therefore, we first conducted a bioinformatics analysis on the putative *bfr* gene and its product Bfr protein. In the genomes of many bacteria, such as *Escherichia coli*, *Pseudomonas aeruginosa*, *Mycobacterium tuberculosis*, and so on, the *bfr* gene is often adjacent to a *bfd* gene, which encodes a bacterioferritin‐associated ferredoxin (Bfd) (Yao *et al*., 2012; McMath *et al*., 2013; Eshelman *et al*., 2017) (Figure 1a). The Bfd protein is suggested to be involved in the delivery of electrons from NADP‐ ferredoxin reductase to haem in the process of iron release from Bfr (Yao *et al*., 2012; Wang *et al*., 2015; Eshelman *et al*., 2017; Punchi Hewage *et al*., 2019). However, the *bfr* gene in the *A. tumefaciens* genome is not adjacent to any *bfd* gene (Figure 1a). This gene organization casts doubt on the annotation of the *bfr* gene in *A. tumefaciens* genome. Bfr protein binds haem by a conserved methionine (M) (Yao *et al*., 2012). To demonstrate if the putative Bfr protein in *A. tumefaciens* has the haem‐binding methionine, we aligned the amino acid sequence of *A. tumefaciens* Bfr with the Bfr sequences from other bacteria (Figure 1b). The sequence alignment shows that M60 might be suitable for binding haem. This confirms that *atu2771* is a *bfr* gene, which encodes a 169 amino acid polypeptide with an extra eight amino acid sequence on the N‐terminal.

The *A. tumefaciens dps* gene (*atu2477*) was cloned and heterologously expressed by *E. coli* in the early 2000s (Ceci *et al*., 2003). The structural and functional properties of *A. tumefaciens* Dps protein were studied in vitro. The N‐terminal tail of *A. tumefaciens* Dps is 11 amino acid residues shorter than in the *E. coli* Dps. A disordered, flexible, and positively charged N‐terminal extension is required for the interaction of Dps protein with DNA, and thus *A. tumefaciens* Dps protein does not bind to DNA in vitro. In vitro experiments showed that *A. tumefaciens* Dps is still able to afford protection of DNA from degradation due to radicals produced in Fenton reactions (Ceci *et al*., 2003). However, the in vivo physiological function of Dps in *A. tumefaciens* remains unclear.

### 2.2 Deletion of two ferritin‐encoding genes affects the growth of *A. tumefaciens* under iron stress

**Figure 2:**
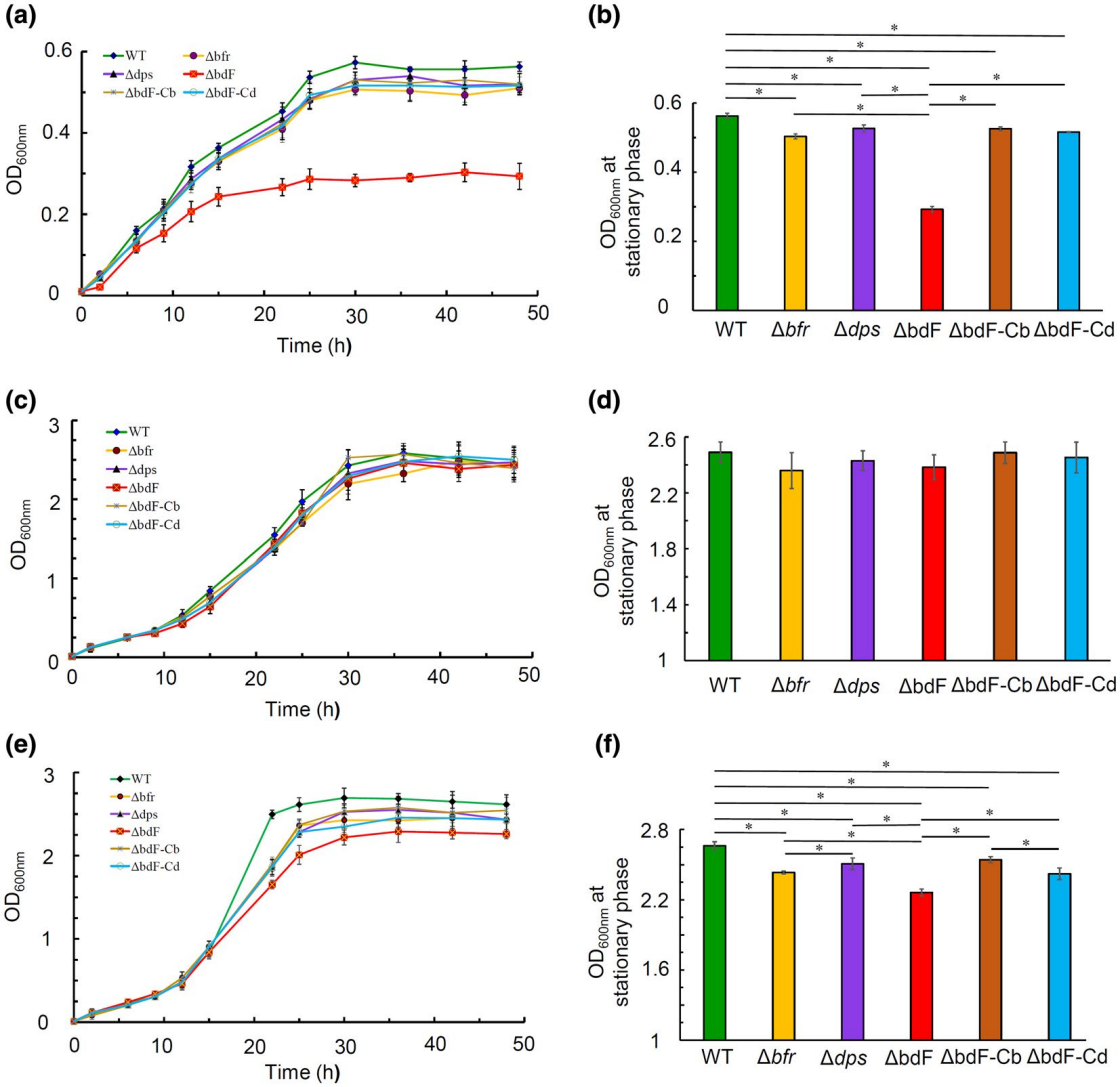
The overall effect of ferritin deficiency on the growth of *Agrobacterium tumefaciens* under various iron concentrations. AB−Fe growth medium supplemented with (a) 2 µM FeSO_4_, (c) 8 µM FeSO_4_, or (e) 20 µM FeSO_4_ were inoculated with the wild type (♦), Δ*bfr* (●), Δ*dps* (▲), ΔbdF (■), ΔbdF‐Cb (*), and ΔbdF‐Cd (○) strains to a final cell density of 10^7^ cfu/ml, respectively. All strains were cultured at 28°C with shaking. Aliquots were taken from the cultures at the indicated time points to measure the cell density. Cell density was expressed by the optical density at 600 nm. Data are the means of three biological replicates with the *SD*. To indicate the statistical significance of the differences among these growth curves, the mean OD_600 nm_ values of the stationary phase (OD_600 nm_ values at time points of 30, 36, 42, and 48 hr) in the growth curves of (a), (c), and (e) are reshown in (b), (d), and (f), respectively. Bars paired by a line with an asterisk are significantly different from one another (*p* < .05 via unpaired Student's *t* test)

To investigate the function of ferritin, two ferritin‐encoding genes (*bfr* and *dps*) in *A. tumefaciens* wild‐type strain C58 were deleted in‐frame using a gene replacement system. Because the main function of ferritin is to store iron and keep iron homeostasis in the cell, the first question we wanted to answer was whether the deficiency of ferritin affects the response of *A. tumefaciens* growth to iron. To precisely control the iron concentration in the medium, a defined medium AB−Fe (AB minimal medium without iron) was used to test the response of *A. tumefaciens* to iron. The wild‐type strain C58, *bfr*‐deletion mutant Δ*bfr*, *dps*‐deletion mutant Δ*dps*, *bfr*‐*dps* double‐deletion mutant ΔbdF, and two complemented strains (strain ΔbdF‐Cb: double mutant ΔbdF was complemented by *bfr*; strain ΔbdF‐Cd: double mutant ΔbdF was complemented by *dps*) were cultured in the AB−Fe media supplemented with different concentrations of iron. The growth curves of six *A. tumefaciens* strains cultured in the AB−Fe media supplemented with different iron concentrations are shown in Figure 2a,c,e. Growth curves show that all strains grew to stationary phase after culture for 30 hr in these media. To demonstrate whether or not the growth differences among these strains are statistically significant, the OD_600nm_ values of every strain at the time points of the stationary phase were used for the unpaired Student's *t* test and the *t* test results are shown in Figure 2b,d,f.

It is obvious that the effects of ferritin on the growth of *A. tumefaciens* depend on the concentration of iron (Figure 2). When the AB−Fe medium was supplemented with 2 μM FeSO_4_, the maximum growth yield of *A. tumefaciens* could only reach an OD_600 nm_ of 0.55 (Figure 2a), demonstrating that the lack of iron severely impedes the growth of all the tested strains. The data in Figure 2a,b show that the OD_600nm_ values of each single mutant at the stationary phase are significantly smaller than those of the wild‐type strain and the double mutant ΔbdF exhibits a more severe growth defect as compared to the single mutants and the wild‐type strain. The growth defect caused by ferritin deficiency can be reversed by the complementation of the corresponding ferritin, implying that iron storage by either Bfr or Dps is important for *A. tumefaciens* to grow under the condition of chronic iron limitation.

When cultured in the AB−Fe medium supplemented with 8 μM FeSO_4_, all three mutants and two complemented strains grew well, as did the wild‐type strain (Figure 2c,d). The 8 μM concentration of FeSO_4_, which is roughly equal to the concentration of iron in the normal AB medium (2.5 mg/L FeSO_4_.7H_2_O in normal AB medium, approximately 9 µM FeSO_4_), is thought to be a moderate iron concentration for *A. tumefaciens* growth. Therefore, results in Figure 2c demonstrate that ferritin is dispensable for *A. tumefaciens* to grow under moderate iron concentration.

Under excessive iron conditions (20 μM FeSO_4_), both growth rate and maximum growth yield of three mutants were smaller than that of wild‐type strain, and especially the *bfr*‐*dps* double‐deletion mutant showed the smallest growth rate and growth yield (Figure 2e,f). The differences among the wild‐type and the three mutants in growth yield are statistically significant and the growth curve of each complemented strain is similar to that of the corresponding single mutant (Figure 2e,f), demonstrating that ferritin is beneficial for the growth of *A. tumefaciens* under relatively high iron concentration. Excessive iron may be harmful to bacterial cells because iron may promote the Fenton reaction, generating highly toxic hydroxyl radicals. Ferritin can mineralize iron, scavenge harmful Fenton reaction (Carrondo, 2003), and thus promote *A. tumefaciens* growth in high iron concentration.

To test whether ferritin deficiency affects *A. tumefaciens* growth in rich medium, we tested the growth curves of all these *A. tumefaciens* strains cultured in yeast extract‐peptone (YEP) medium (Gelvin, 2006). Growth curves showed that the deletions of *bfr* and *dps* genes do not affect the growth of *A. tumefaciens* in YEP medium (data not shown), indicating that ferritin protein is also dispensable for the growth of *A. tumefaciens* if it grows in rich medium. Thus, the results in Figure 2 allow us to conclude that ferritin is required for *A. tumefaciens* growth only under iron stress (both iron limitation and excess).

### 2.3 Ferritin deficiency significantly affects the intracellular iron level of *A. tumefaciens* and two ferritin proteins have cumulative effects on the accumulation of iron in cells

**Figure 3:**
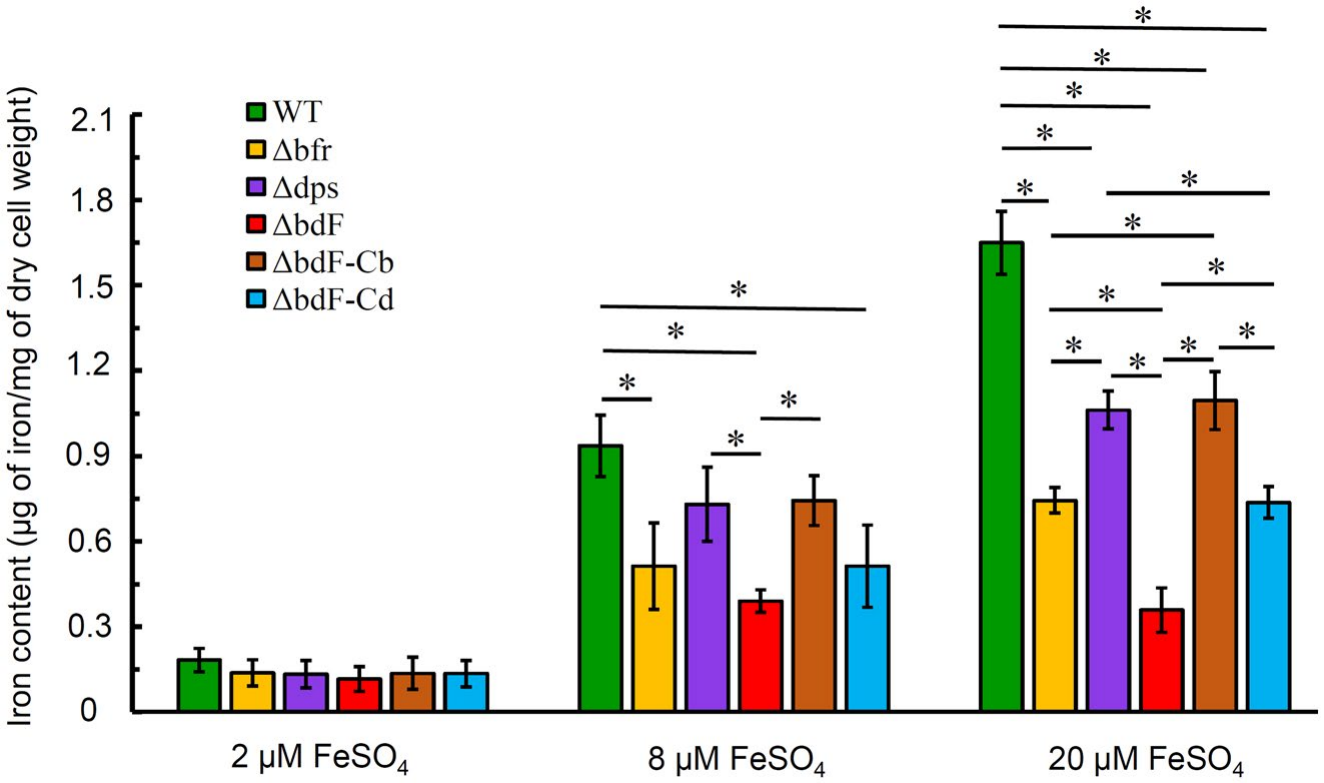
The overall effect of ferritin deficiency on the total intracellular iron content of *Agrobacterium tumefaciens* cultured under various iron concentrations. Wild‐type (WT, green bars), Δ*bfr* (yellow bars), Δ*dps* (purple bars), ΔbdF (red bars), ΔbdF‐Cb (orange bars), and ΔbdF‐Cd (blue bars) strains were grown to late log phase in AB−Fe growth medium supplemented with 2 µM, 8 µM, or 20 µM FeSO_4_. Agrobacterial cells were collected from the cultures and washed four times (twice with buffer containing 10 mM EDTA and twice with deionized water). Total amounts of iron were measured by inductively coupled plasma‐optical emission spectrometry. Data are the means of three biological replicates with the *SD*. Bars paired by a line with an asterisk are significantly different from one another (*p* < .05 via unpaired Student's *t* test)

To assess the effects of ferritin deficiency on the cellular iron content of *A. tumefaciens*, three mutants (Δ*bfr*, Δ*dps*, and ΔbdF), two complemented strains (ΔbdF‐Cb and ΔbdF‐Cd), and the wild type of *A. tumefaciens* were grown to the late log phase in defined medium supplemented with different concentrations of FeSO_4_. The total intracellular iron content in these mutant and wild‐type cells was analysed using inductively coupled plasma‐optical emission spectrometry (ICP‐OES). The results are shown in Figure 3.

Under moderate (8 μM FeSO_4_) and excessive (20 μM FeSO_4_) iron conditions, the total intracellular iron content of each mutant was lower than that of the wild type and the Student's *t* test showed that the differences of intracellular iron content between these compared strains were statistically significant. In particular, the double mutant ΔbdF contained approximately 4.5‐fold less iron than the wild type in the cells when grown under excessive iron conditions. Additionally, complementation by either *bfr* or *dps* restored the total intracellular iron content of the double mutant ΔbdF to the level of the corresponding single mutant (Figure 3).

Although the intracellular iron contents of all three mutants (Δ*bfr*, Δ*dps*, and ΔbdF) grown in the iron‐limited medium (2 μM FeSO_4_) were not statistically different from the wild type (Figure 3), it can still be concluded that ferritin significantly affects the accumulation of iron in *A. tumefaciens* cells. Our explanation for this indifference is that the iron‐limited media are unable to provide enough iron for *A. tumefaciens*. The results also show that Bfr and Dps have cumulative effects on the accumulation of iron in cells. Furthermore, Bfr was more beneficial for the accumulation of iron than Dps because the *bfr* mutant contained less iron than the *dps* mutant (Figure 3).

### 2.4 Deletion of two ferritin‐encoding genes impairs the resistance of *A. tumefaciens* to oxidative stress

It was reported that ferritins can protect many bacteria against oxidative stress (Boughammoura *et al*., 2008; Oliveira *et al*., 2020). To determine if the deletion of ferritin‐encoding genes affected the oxidative stress survival of *A. tumefaciens*, the survival rate of agrobacterial cells after short‐term exposure to high H_2_O_2_ concentration was used to evaluate the resistance of *A. tumefaciens* to oxidative stress.

**Figure 4:**
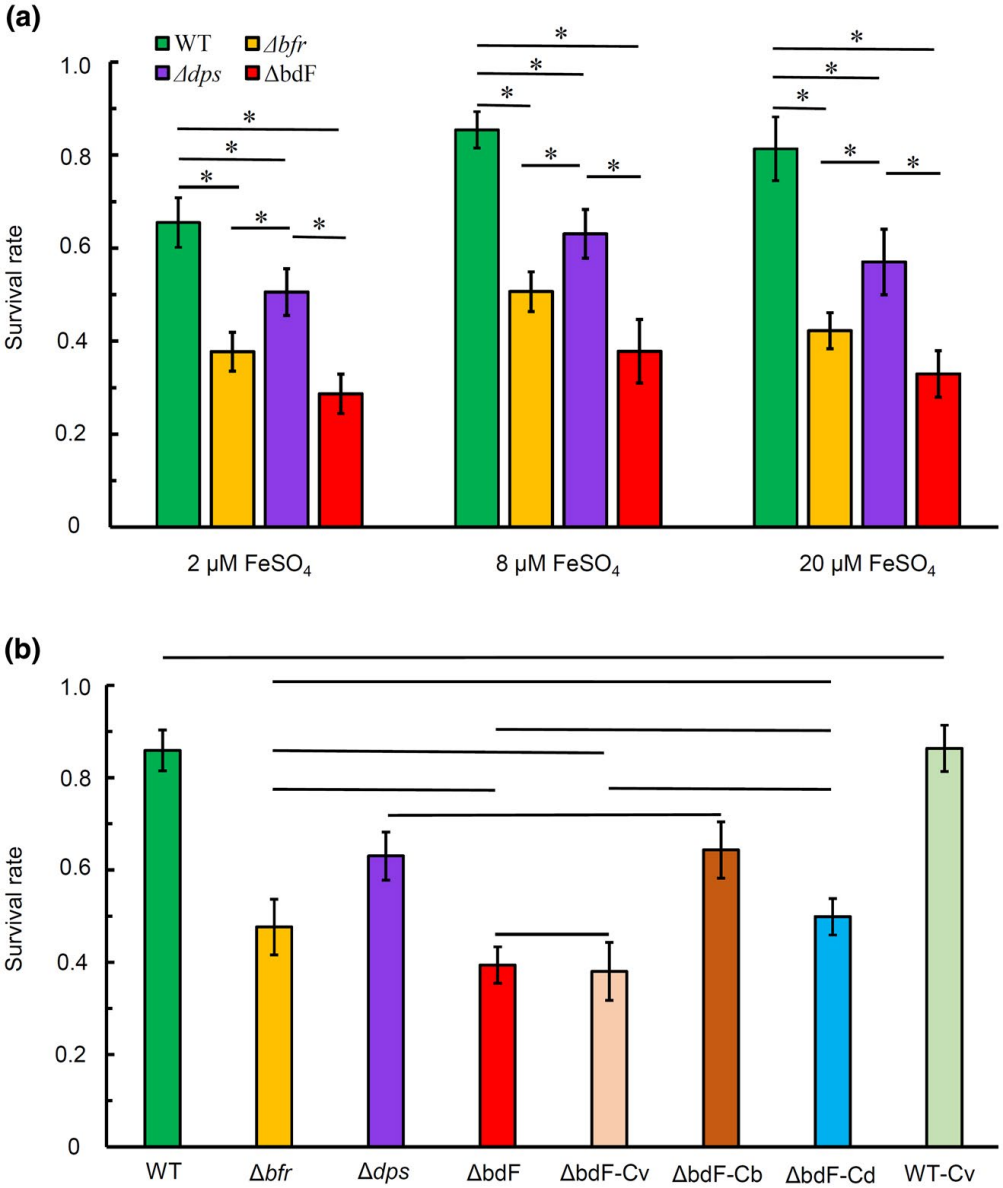
Effects of ferritin deficiency on the resistance of *Agrobacterium tumefaciens* to H_2_O_2_.Various *A. tumefaciens* strains were grown to mid‐log phase in (a) AB−Fe medium supplemented with either 2 µM, 8 µM, or 20 µM FeSO_4_ and (b) normal AB medium (roughly 9 µM FeSO_4_). Agrobacterial cells were collected from these cultures and washed with AB−Fe liquid medium twice. The washed cells of all these tested strains were resuspended in AB−Fe medium to an OD_600 nm_ of 0.2. The cell suspensions were treated in the AB−Fe medium containing 3 mM H_2_O_2_ at 25°C with shaking for 0.5 hr. After treatment, equal amounts of cells from treated or untreated cell suspensions were used to count cfus. The cfu ratio of the treated cell suspension to the untreated cell suspension was used to define the survival rate. Data are the means of three biological replicates with the *SD*. (a) Bars paired by a line with an asterisk are significantly different from one another (*p* < .05 via unpaired Student's *t* test). (b) Except for the bars paired by a line, all other bars are significantly different from each other (*p* < .05 via unpaired Student's *t* test). WT, wild‐type *A. tumefaciens* C58; Δ*bfr*, *bfr* deletion mutant; Δ*dps*, *dps* deletion mutant; ΔbdF, *bfr* and *dps* double‐deletion mutant; ΔbdF‐Cb, ΔbdF mutant complemented with *bfr* gene; ΔbdF‐Cd, ΔbdF mutant complemented with *bfr* gene; WT‐Cv, C58 wild‐type bearing empty vector; ΔbdF‐Cv, ΔbdF bearing empty vector

As shown in Figure 3, both the iron concentration in the medium and the ferritin in the cell affect the intracellular iron level of *A. tumefaciens*. One of the major mechanisms of ferritins protecting bacteria against oxidative stress is regulating the concentration of free intracellular iron (Touati, 2000). Accordingly, the wild type and three mutants (Δ*bfr*, Δ*dps*, and ΔbdF) were precultured in AB−Fe medium supplemented with different concentrations of FeSO_4_ before these strains were used to test their resistance to H_2_O_2_. Because free Fe^2+^ in the medium will result in the rapid catalytic decomposition of H_2_O_2_ via the Fenton reaction, the agrobacterial cells, which were precultured in AB−Fe medium supplemented with different concentrations of FeSO_4_, were washed with the iron‐free AB−Fe medium twice and then treated with 3 mM H_2_O_2_ in AB−Fe medium for 0.5 hr. Both the treated and untreated cells were used to count cfus. The cfu ratio of treated cells to untreated cells was used as a measure of the survival rate, representing the viability of the treated cells. As shown in Figure 4a, after exposure to 3 mM H_2_O_2_ for 0.5 hr, all three ferritin mutants (Δ*bfr*, Δ*dps*, and ΔbdF) showed a significant decrease in viability in comparison to the wild type no matter what level of iron was used as the supplement in the preculture medium, demonstrating that ferritin deficiency impairs the resistance of *A. tumefaciens* to oxidative stress. Figure 4a also shows that the double mutant had the lowest survival rate and the survival rate of *bfr* mutant was less than that of *dps* mutant. Based on the survival rate, it appeared that *bfr* and *dps* had a joint additive effect in protecting agrobacterial cells against the H_2_O_2_ oxidative stress.

To further verify the effects of ferritin deficiency on the oxidative resistance of agrobacterial cells, the H_2_O_2_ resistance of the wild type and three mutants (Δ*bfr*, Δ*dps*, and ΔbdF) was retested along with two complemented strains (ΔbdF‐Cb and ΔbdF‐Cd) and two control strains (WT‐Cv: wild type bearing empty vector; ΔbdF‐Cv:ΔbdF bearing empty vector). All these strains were precultured in normal AB medium and treated with 3 mM H_2_O_2_ in the iron‐free AB−Fe medium for 0.5 hr. As shown in Figure 4b, the complementation of each ferritin restored the survival rate of the double mutant ΔbdF to the level of the corresponding single mutant, and the addition of empty vector to either wild type or double mutant ΔbdF did not affect the survival rate, confirming that ferritin can protect *A. tumefaciens* against oxidative stress.

### 2.5 Deletion of two ferritin‐encoding genes attenuates the tumourigenicity of *A. tumefaciens*

The deletion of two ferritin‐encoding genes affects not only the growth of *A. tumefaciens* under both limited and excessive iron conditions but also its susceptibility to oxidative stress in minimal media. However, it remains unknown if ferritin deficiency affected the pathogenicity of *A. tumefaciens*. In order to test the possible effect of ferritin deficiency on *A. tumefaciens* virulence and to determine if the effect of ferritin deficiency on *A. tumefaciens* virulence is related to the oxidative stress condition, the pathogenicity of wild type (C58), ferritin‐deficient mutant strains (Δ*bfr*, Δ*dps*, and ΔbdF), and complemented strains (ΔbdF‐Cb and ΔbdF‐Cd) of *A. tumefaciens* was tested under two different infection conditions: kalanchoe leaves with photosynthesis and potato tuber without photosynthesis. All tested *A. tumefaciens* strains were precultured in AB−Fe medium supplemented with different iron concentrations or AB minimal medium to mid‐log phase. The OD_600 nm_ values of each culture were converted to cfu in a pilot experiment. Equal amounts of cells (cfu) from different cultures were inoculated onto the wound sites of kalanchoe leaves and potato tuber discs.

**Figure 5:**
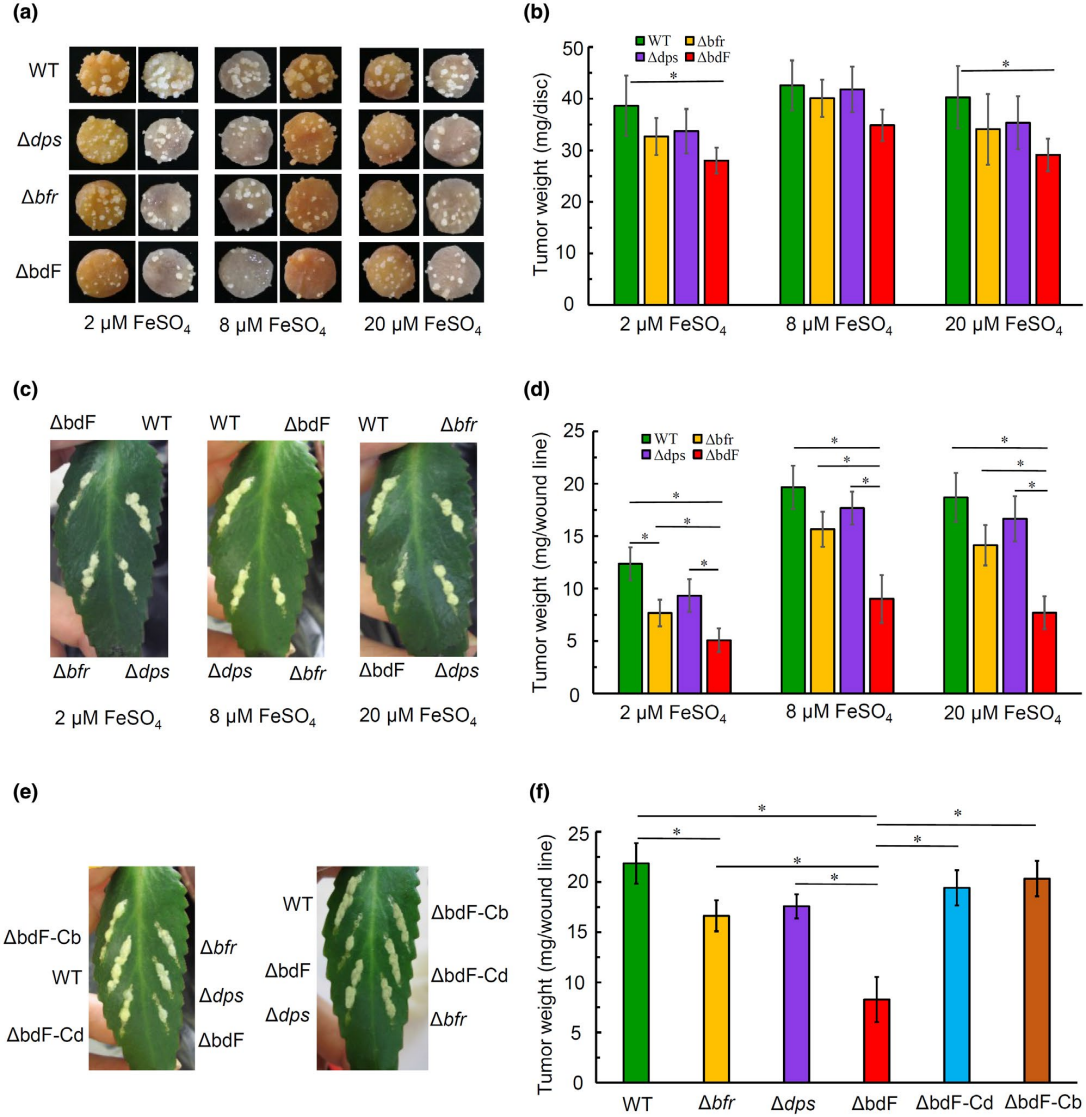
Ferritin is required for the full virulence of *Agrobacterium tumefaciens*. The tested strains were precultured to mid‐log phase in AB−Fe medium supplemented with 2 µM, 8 µM, or 20 µM FeSO_4_. In (e) and (f), the tested strains were precultured in AB medium (roughly 9 µM FeSO_4_). Agrobacterial cells were collected from the cultures and washed twice. Cell densities of all the tested strains were adjusted to 10^9^ cfu/ml. Two microlitres of cell suspension (2 × 10^6^ cfu) was inoculated onto each potato tuber disc (a) or each wound line on kalanchoe leaves (c, e). The representative tumours in (a), (c), and (e) were photographed 28–35 days after inoculation. To quantify the pathogenicity, tumours were carefully scraped from potato tuber discs or kalanchoe leaves, and weighed (b, d, f). The inoculated strain is identified on the left (a), four corners (c), or both sides (e) of the photographs. WT, wild‐type strain C58; Δbfr, *bfr* deletion mutant; Δdps, *dps* deletion mutant; ΔbdF, *bfr*‐*dps* double‐deletion mutant; ΔbdF‐Cb, Δbdf strain complemented Bfr by plasmid; ΔbdF‐Cd, Δbdf strain complemented Dps by plasmid. Data are the means of three biological replicates with the standard deviation. Pairs of bars that are significantly different from one another are indicated by a line with an asterisk (*p* < .05 via unpaired Student's *t* test)

Photographs of typical tumours on potato tuber discs are shown in Figure 5a. It was difficult to determine the differences between the tumours on the potato discs from photographs, so we scraped the tumours from the potato discs and weighed the tumour weight. The fresh weight of the tumours induced by each strain is shown in Figure 5b. The weight of tumours induced by each mutant (Δ*bfr*, Δ*dps*, or ΔbdF) on potato discs was slightly less than that induced by the wild type. Only the *bfr‐dps* double mutant ΔbdF, which was precultured in the AB−Fe medium supplemented with 2 µM and 20 µM FeSO_4_, induced significantly less tumour weight than the wild type did. These results did not discourage us. We used kalanchoe leaves as the host and let *A. tumefaciens* induce tumours under natural conditions. From the typical tumour photographs in Figure 5c, it can be observed that tumour induced by the double mutant ΔbdF was much less than that induced by any other strains whatever medium these strains were precultured in. The data in Figure 5d affirm that the tumour weight induced by the *A. tumefaciens* strain without any ferritin (ΔFtn) was significantly less than that induced by any other tested strains. Furthermore, agrobacterial cells that were precultured in the iron‐limited medium induced much less tumour than other cells did.

To further verify the role of ferritin in the tumorigenesis of *A. tumefaciens*, the effects of the complementation of either Bfr or Dps on the tumourigenicity of the double mutant ΔbdF were examined. As shown in Figure 5e,f, tumour weight induced by the complemented strain (ΔbdF‐Cb or ΔbdF‐Cd) was similar to that induced by the corresponding single mutant strain, demonstrating that the complementation of ferritin restored the defective ability of the ferritin‐deficient strain to induce tumours. Therefore, ferritin plays an important role in the tumorigenesis of *A. tumefaciens*. If we further compare the tumour weight induced on two different hosts by each strain, we may conclude that the impact of ferritin deficiency on *A. tumefaciens* virulence varies with host and/or tumour‐inducing condition.

## 3 DISCUSSION

In this study, we explored the effects of ferritin on the cell growth, oxidative stress tolerance, and virulence of *A. tumefaciens*. By testing the tumorigenesis of *A. tumefaciens* ferritin‐deficient mutants on plants, our results demonstrated that the deficiency of Bfr ferritin severely attenuates the tumour formation by *A. tumefaciens*.

Previous studies have demonstrated that bacteria can use both Bfr and Ftn to store iron (Boughammoura *et al*., 2008). However, some bacteria, which have both Bfr and Ftn, prefer Ftn to store iron. For example, *Erwinia chrysanthemi*, a pathogenic enterobacterium that is able to cause soft rot diseases in many crops, possesses both the FtnA‐encoding and Bfr‐encoding genes. A *bfr*‐deficient mutant of *E. chrysanthemi* showed no obvious phenotypic difference from the wild type. Nevertheless, the deficiency of FtnA significantly affects the iron storage and metabolism of *E. chrysanthemi* (Abdul‐Tehrani *et al*., 1999; Boughammoura *et al*., 2008). *A. tumefaciens* does not have an Ftn‐encoding gene, so the choice for iron storage is Bfr or Dps. Our results verified that both Bfr and Dps play an important role in the iron storage, homeostasis, and metabolism of *A. tumefaciens*, but Bfr stores more iron than Dps (Figure 3).

Fluctuation of iron availability is a critical environmental stress for many pathogens during the infection of hosts because hosts often compete with invading microbes for iron. As a result, the acquisition of iron is essential for phytopathogens to survive in the host (Ratledge and Dover, 2000; Yu and Ye, 2016). Bacterioferritin, which acts as a major iron store, plays a key role in intracellular iron distribution, particularly under iron‐limited conditions (Expert *et al*., 2008; Lemanceau *et al*., 2009). According to the response of the ferritin‐deficient mutant of *A. tumefaciens* to iron, ferritin is able to store iron for *A. tumefaciens* to grow when *A. tumefaciens* can get a plentiful supply of iron (Figure 3). An iron reserve in cells before invading a host may be important for the pathogenicity of *A. tumefaciens*. When precultured in AB minimal medium before inoculation, ferritin‐deficient mutants of *A. tumefaciens* are unable to store much iron. The low level of iron reserves in cells may be one of the key factors that result in much less tumour induced by ferritin‐deficient mutants (Figure 5).

After infection by pathogenic microbes, host plants often generate an oxidative burst as a barrier of resistance to pathogen attack (Fagard *et al*., 2007). For the infection of photosynthetic tissues, an oxidative environment caused by photosynthesis is also an oxidative stress to phytopathogen. Thus, an oxidative burst is another critical environmental stress, and it is essential for phytopathogens to cope with the reactive oxygen species from environments, including that produced by host plant cells. Several studies on *E. chrysanthemi* have demonstrated the importance of ferritins to maintain iron homeostasis or a connection between iron metabolism and oxidative stress tolerance (Nachin *et al*., 2001; Okinaka *et al*., 2002; Yang *et al*., 2004). In *A. tumefaciens*, ferritin also plays an important role in resisting oxidative stress (Figure 4). The reduction in oxidative stress resistance caused by ferritin deficiency may be one of the reasons that ferritin affects the pathogenicity of *A. tumefaciens*.

## 4 EXPERIMENTAL PROCEDURES

### 4.1 Bacterial strains, plasmids, and growth conditions

The strains and plasmids used in the study are listed in Table S1. DNA molecular manipulations for constructing plasmids followed standard molecular protocols and DNA molecules were introduced into *E. coli* cells by heat shock (Sambrook *et al*., 1989). *E. coli* strains were routinely used as the host for DNA cloning and cultured in lysogeny broth (LB) liquid or agar medium at 37°C. Plasmids were transferred into *A. tumefaciens* by electroporation (Cangelosi *et al*., 1991). *A. tumefaciens* strains were cultured in two different media (rich medium YEP and minimal medium AB) at 28°C. YEP and AB media were prepared according to previous references (Gelvin, 2006; Guo *et al*., 2007a; Yang *et al*., 2015). Corresponding antibiotics were used to culture different bacterial strains as required. The concentrations of antibiotics used for different bacteria were the same as described in previous reports(Guo *et al*., 2007a; Huang *et al*., 2018).

To easily adjust the concentration of iron in the medium, FeSO_4_ prescribed in AB minimal medium was omitted. AB medium without iron was called AB−Fe medium in this study. Both AB and AB−Fe media were prepared with deionized water. To test the effect of iron on the growth of different *A. tumefaciens* strains, *A. tumefaciens* cells from the YEP culture were washed two times with AB−Fe medium and then cultured in AB−Fe medium supplemented with the iron chelator 2,2′‐dipyridyl (200 µM) for 1 hr so that the plentiful iron in bacterial cells was depleted. Bacterial cells without plentiful iron were washed two times with AB−Fe medium, adjusted to the same cell density and then transferred to AB−Fe medium supplemented with different concentrations of iron, as indicated in each experiment. A pilot experiment was carried out for each strain to convert OD_600 nm_ to cfu. To measure the growth curve, each *A. tumefaciens* strain was inoculated to the growth medium with an initial cell density of 10^7^ cfu/ml. The growth rates of different *A. tumefaciens* strains in the media with different concentrations of iron were monitored by measuring OD_600 nm_ at given time intervals. The growth rates were tested in three biological replicates and the mean ± *SD* was used to plot the growth curves.

### 4.2 Construction of gene‐deficient mutants and complemented strains

Because the genome of *A. tumefaciens* C58 has been sequenced (genome accession number: AE007869.2) (Goodner *et al*., 2001), the precise deletion of *A. tumefaciens* gene *bfr* can be constructed by homologous recombination according to previous procedures (Guo *et al*., 2007b, 2009; Huang *et al*., 2018). Plasmid pEX18Km was used as the gene replacement vector. It is unable to replicate in *A. tumefaciens* and carries a kanamycin‐resistance gene, which was used as the positive selection marker to select for single cross‐over colonies, and a levansucrase gene (*sac*B), which was used as the negative selection marker to select for double cross‐over colonies (Guo *et al*., 2009). The locus tag for *bfr* gene (510 bp) is *atu2771*, which located at position 2,776,477 to 2,776,986 on the *A. tumefaciens* C58 genome. The 5′ flank (886 bp) and 3′ flank (1,021 bp) of the *bfr* gene were amplified by PCR (the primer sequences are listed in Table S2). These two sequences were assembled into a 1,907 bp fragment by overlapping PCR. This 1,907 bp fragment, which contains the flank sequences of *bfr* but not the 510‐bp *bfr* coding sequence, was cloned into plasmid pEX18Km through *Bam*HI and *Hin*dIII sites to obtain the plasmid pEX18Km‐bfr for *bfr* deletion. This plasmid was transformed into *A. tumefaciens* C58 by electroporation. The *bfr* deletion mutants were identified among the double cross‐over colonies by PCR and further confirmed by DNA sequencing (Figure S1a).

To precisely delete the *dps* gene, the 901‐bp upstream and 1,021‐bp downstream sequences of *dps* without the 486‐bp *dps* coding sequence were assembled into a 1,922 bp fragment by overlapping PCR. This 1,922 bp fragment was inserted into plasmid pEX18Km through *Bam*HI and *Hin*dIII sites to generate plasmid pEX18Km‐dps for *dps* deletion. This plasmid was introduced to *A. tumefaciens* cells. The *dps*‐deficient mutant was generated via homologous recombination and was identified as the aforementioned procedure for constructing the *bfr* deletion mutant (Figure S1b,c).

The complemented strains of ferritin‐deficient mutants were constructed by introducing a ferritin‐expressing plasmid to the ferritin‐deficient mutants. To construct the ferritin‐expressing plasmids, a DNA fragment comprising the intact gene (*dps* or *bfr* gene) and its native promoter (roughly 500 bp upstream of the start code) was amplified through PCR from *A. tumefaciens* genomic DNA and was cloned into plasmid pCB301. Two complementing plasmids, pCB301‐dps and pCB301‐bfr, were constructed. Plasmid pCB301‐dps was used to complement ferritin Dps and plasmid pCB301‐bfr was used to complement ferritin Bfr.

### 4.3 Determination of total cellular iron content

*A. tumefaciens* strains were grown in AB−Fe medium supplemented with the indicated concentrations of iron. Cells were harvested at late log phase by centrifugation (c.3,800 × g, 10 min). The cells were washed twice with 50 mM phosphate‐buffered saline (PBS, pH 6.0) containing 10 mM EDTA. To remove excess ion, the cells were washed twice with deionized water and then resuspended in deionized water to achieve an OD_600 nm_ of 1. The cell suspension was divided into two parts. One part was used to determine the dry weight of the cells in the cell suspension. The other part of the cell suspension was digested with 70% ultrapure nitric acid at 98°C for 3 hr and then was used for the iron content determination. The iron content in each sample was determined by ICP‐OES (Optima 7300 DV, PerkinElmer). The data are presented as the mean and *SD* of three biological replicates.

### 4.4 H_2_O_2_ sensitivity tests

The sensitivities of different *A. tumefaciens* strains to H_2_O_2_ were evaluated by the survival rate of agrobacterial cells after treatment with 3 mM H_2_O_2_ for 0.5 hr. Various *A. tumefaciens* strains were cultured to mid‐log phase in AB−Fe medium supplemented with different concentrations of iron or in normal AB medium. Cells were collected, washed twice with AB−Fe medium, and resuspended in AB−Fe medium. Cell suspensions of all the tested strains were normalized to an OD_600 nm_ of 0.2 with AB−Fe medium and divided into two parts: one part was treated with 3 mM H_2_O_2_ in the AB−Fe medium, the other part was left untreated. After the cell suspensions were treated in the AB−Fe medium containing 3 mM H_2_O_2_ at 25°C with shaking for 0.5 hr, both the treated and untreated cell suspensions were used to count cfu. The cell survival rate of the treated cell suspension was measured as the cfu ratio of the treated cell suspension to the untreated cell suspension. All these tests were performed in three biological replicates on separate days and the data are presented as the mean with *SD*.

### 4.5 Tumorigenesis assays

Two hosts (potato tuber and kalanchoe leaf) were used for the tumorigenesis assays. The tumorigenesis assay in the potato (*Solanum tuberosum*) tuber was adapted a previously described method (Anand and Heberlein, 1977). Pilot experiments were conducted for all strains cultured in different media to adjust OD_600 nm_ to cfu. *A. tumefaciens* strains were grown to an OD_600 nm_ of 0.4–0.6 in AB−Fe medium supplemented with different concentrations of iron. Cells were harvested via centrifugation, washed twice with PBS, and then resuspended in PBS at 10^9^ cfu/ml for inoculation. Potato tubers of moderate size were peeled and surface sterilized by immersing in 1.05% sodium hypochlorite for 30 min. A tuber cylinder was extracted from the core of each potato tuber with a surface sterilized, 2‐cm diameter cork borer and then sliced into 5 mm thick discs with a sterile scalpel. The potato tuber discs were transferred to 1.5% water agar plates and each potato tuber disc was inoculated with 2 μl of agrobacterial cell suspension (2 × 10^6^ cfu). The infected potato tuber discs were incubated at 25°C for 4–5 weeks. To determine the fresh weight of galls from each potato tuber disc, tumours were carefully scraped from the discs using a scalpel to minimize contamination of potato tuber tissue and weighed on a precision balance.

The tumorigenesis assay in the kalanchoe leaf was carried out as described before (Guo *et al*., 2007a,2007b). Agrobacterial cells for the infection to kalanchoe leaf were prepared as for the infection to potato tuber. Kalanchoe plants were naturally grown in pots. Kalanchoe leaves in the leaf age of 2–3 weeks were chosen for *Agrobacterium* infection. To eliminate the possible effect of leaf age on tumorigenesis, all the compared *Agrobacterium* strains were inoculated onto the same leaf. In each kalanchoe leaf, several wound lines were made using a hypodermic needle. Each wound line was inoculated with one of the *Agrobacterium* strains. The wound lines on the same leaf were randomly allotted to different *Agrobacterium* strains. Each wound line was inoculated with 2 μl of agrobacterial cell suspension (2 × 10^6^ cfu). The infected kalanchoe leaves were allowed to grow naturally for 30–35 days. Tumours in the infected wound lines were carefully scraped and weighed on a balance.

Experiments were repeated in three biological replicates on separate days and nine potato tuber discs or nine kalanchoe leaves were assayed for each treatment in these three biological replicates. The data are presented as the mean and *SD* of three biological replicates.

### 4.6 Statistical analysis

The Microsoft Office Excel data analysis tool was used to perform the statistical analysis. An unpaired Student's *t* test was performed to assess the statistical difference between the measurements. A value of *p* < .05 was considered statistically significant.

## Supporting information


**FIGURE S1**

**TABLE S1**

**TABLE S2**

## Data Availability

The data that support the findings of this study are available from the corresponding author upon reasonable request.
