# Supplementary material for: Agrobacterium tumefaciens ferritins play an important role in full virulence through regulating iron homeostasis and oxidative stress survival
Source: Mol Plant Pathol. 2020 Jul 17;21(9):1167–78. doi: 10.1111/mpp.12969 (PMC7411545; doi:10.1111/mpp.12969)
Supplement: Supplementary file 1 — FIGURE S1 [file MPP-21-1167-s001.docx]

**Figure S1** Identification of *bfr*-deletion mutant Δ*bfr*, *dps*-deletion mutant Δ*dps*, and *bfr*-*dps* double-deletion mutant ΔbdF. (a) To generate *bfr*-deletion mutant, plasmid pEX18Km-bfr was introduced into *A. tumefaciens* wild type strain C58. The single cross-over transformant, which was generated by the integration of the whole plasmid via intermolecular homologous recombination occurring at either flank of *bfr* gene, was selected on kanamycin-containing MG/L agar plate and verified by PCR. The purified and verified single cross-over transformant was plated on sucrose-containing MG/L plate to select the double cross-over recombinant, which was generated by the deletion of plasmid backbone (or together with *bfr* gene) via the intramolecular homologous recombination occurring between two repeated flanks of *bfr* gene. Therefore, two types of the double cross-over recombinant were generated: one reverted to the wildtype and the other was the *bfr*-deletion mutant. For the detailed principal and selecting procedure of this gene replacement protocol, please consult the reference of Guo *et al* (2009). These two types of recombinant were identified by PCR using the primers bfr-f1 and bfr-r2. The bacterial cells from different double cross-over colonies and plasmid pEX18Km-bfr were used as the PCR templates. The PCR products were analyzed by agarose electrophoresis. If the recombinant reverts to the wildtype, the PCR fragment will be 2.417 kb, which includes 5′ flank (886 bp), orf (510 bp) and 3′ flank (1021 bp) of *bfr* gene. If the recombinant is the *bfr*-deletion mutant, the PCR fragment will be 1.907 kb, which includes only the 5′ flank (886 bp) and 3′ flank (1021 bp) of *bfr* gene. In (a), colonies 1, 3, 5, 8, 9, 13, 14, 16, 17, 19, and 20 are *bfr*-deletion mutant; colonies 4, 6, 10, 11, 15, and 18 revert to wildtype; colonies 2 and 7 are not single colony. To generate *dps*-deletion mutant and *bfr*-*dps* double-deletion mutant, plasmid pEX18Km-dps was introduced into *A. tumefaciens* wild type strain C58 (b) and *bfr*-deletion mutant (c) respectively. Primers dps-f1 and dps-r2 were used for the PCR to identify *dps*-deletion mutant (b) and *bfr*-*dps* double-deletion mutant (c). PCR fragment from *dps*-wildtype is 2.408 kb. PCR fragment from *dps*-deletion mutant is 1.922 kb. In (b), colonies 1, 6, 15, and 16 are *dps*-deletion mutants. In (c), colonies 14 and 21 are *bfr*-*dps* double-deletion mutant. The PCR fragments from mutants were further verified by DNA sequencing.

**REFERENCE**

Guo, M., Zhu, Q. and Gao, D. (2009) Development and optimization of method for generating unmarked *A. tumefaciens* mutants. *Prog. Biochem. Biophys*. 36, 556-565.
