# Supplementary material for: Agrobacterium tumefaciens ferritins play an important role in full virulence through regulating iron homeostasis and oxidative stress survival
Source: Mol Plant Pathol. 2020 Jul 17;21(9):1167–78. doi: 10.1111/mpp.12969 (PMC7411545; doi:10.1111/mpp.12969)
Supplement: Supplementary file 2 — TABLE S1 [file MPP-21-1167-s002.docx]

Table S1 Bacterial strains and plasmids used in this study.

| **Strains or plasmids** | **Description** | **Source** |
| --- | --- | --- |
| **Strains** |  |  |
| *Escherichia coli* |  |  |
| DH5α | *endA1 hsdR17 supE44 thi-1 recA1 gyrA96 relA1 (argF-lacZYA) U169 φ80dlacZ.* | Bethesda Research Laboratories |
| *Agrobacterium tumefaciens* |  |  |
| C58 | Wild-type, nopaline-type pTiC58 plasmid | Thomashow et al., 1980. |
| △*bfr* | Derivative of C58 in which *bfr* was deleted | This study |
| △*dps* | Derivative of C58 in which *dps* was deleted | This study |
| ΔbdF | Derivative of C58 in which both *bfr* and *dps* were deleted | This study |
| ΔbdF-Cb | Complemented strain of △bdF by *bfr* gene harbored on plasmid pCB301-bfr | This study |
| ΔbdF-Cd | Complemented strain of △bdF by *dps* gene harbored on plasmid pCB301-dps | This study |
| WT-Cv | C58 bearing empty vector pCB301 | This study |
| ΔbdF-Cv | ΔbdF bearing empty vector pCB301 | This study |
| **Plasmids** |  |  |
| pEX18Km | Gene replacement vector carrying a counter selectable marker *sacB*, *oriT*, Km^R^ | Hoang et al. 1998; Guo et al. 2007 |
| pEX18Km-bfr | pEX18Km carrying the upstream and downstream fragments of *bfr* gene, Km^R^; for the deletion of *bfr* gene | This study |
| pEX18Km-dps | pEX18Km carrying the upstream and downstream fragments of *dps* gene, Km^R^; for the deletion of *dps* gene | This study |
| pCB301 | A mini binary vector plasmid, with MCS from pBI101, Km^R^ | Xiang et al. 1999 |
| pCB301-bfr | pCB301 carrying *bfr* gene and its native promoter sequence, Km^R^ | This study |
| pCB301-dps | pCB301 carrying *dps* gene and its native promoter sequence, Km^R^ | This study |

Km^R^ = Resistant to kanamycin.

**REFERENCES**

Hoang, T.T., Karkhoff-Schweizer, R.R., Kutchma, A.J. and Schweizer, H.P. (1998) A broad-host-range Flp-FRT recombination system for sites pecific excision of chromosomally-located DNA sequences: Application for isolation of unmarked *Pseudomonas aeruginosa* mutants. *Gene* 212, 77-86.

Guo, M., Hou, Q.M., Hew, C.L. and Pan, S.Q. (2007) *Agrobacterium* VirD2-binding protein is involved in tumorigenesis and redundantly encoded in conjugative transfer gene clusters. *Mol. Plant Microbe Interact*. 20, 1201-1212.

Thomashow, M.F., Nutter, R., Montoya, A.L., Gordon, M.P. and Nester, E.W. (1980) Integration and organization of Ti plasmid sequences in crown gall tumors. *Cell* 19, 729-739.

Xiang, C., Han, P., Lutziger, I., Wang, K. and Oliver, D.J. (1999) A mini binary vector series for plant transformation. *Plant Mol. Biol*. 40:711-717.
