# Supplementary material for: Agrobacterium tumefaciens ferritins play an important role in full virulence through regulating iron homeostasis and oxidative stress survival
Source: Mol Plant Pathol. 2020 Jul 17;21(9):1167–78. doi: 10.1111/mpp.12969 (PMC7411545; doi:10.1111/mpp.12969)
Supplement: Supplementary file 3 — TABLE S2 [file MPP-21-1167-s003.docx]

Table S2 Primers used in this study.

| **Primers** | **Sequence** | **Description** |
| --- | --- | --- |
| bfr-f1 | 5' CGCGGATCCGCATCGTCAACATTTCCTCCATCA 3' | To amplify the upstream fragment of *bfr* |
| bfr-r1 | 5' CTGGCGCCAGTGCGGAATCGCTTCAAGC 3' | Over-lapping primer; to amplify the upstream fragment of *bfr* |
| bfr-f2 | 5' TTGAAGCGATTCCGCACTGGCGCCAGAACGA 3' | Over-lapping primer; to amplify the downstream fragment of *bfr* |
| bfr-r2 | 5'CCCAAGCTTTCACCAATTATACCGGC GTCATGA3' | To amplify the downstream fragment of *bfr* |
| dps-f1 | 5'CGCGGATCCGATCATCGCCAGTCAGATCAGCAA3' | To amplify the upstream fragment of *dps* |
| dps-r1 | 5'AAATTCTTCCGGCCGATGTCTCCTGAAGTTG 3' | Over-lapping primer; to amplify the upstream fragment of *dps* |
| dps-f2 | 5' TCAGGAGACATCGGCCGGAAGAATTTCCAAC 3' | Over-lapping primer; to amplify the downstream fragment of *dps* |
| dps-r2 | 5' CCCAAGCTTTGCAGCTTCCCGTCTTCATGGTAA 3' | To amplify the downstream fragment of *dps* |
| HBbfr-f | 5' CGCGGATCCCGGCCCGACGCTGATGATAAACTT TTCGTGATAAT 3′ | To amplify *bfr* gene for complementation |
| HBbfr-r | 5' CCCAAGCTTTTATTCCGTTCGTTGGCAGAATCGG CGTTGAGCT 3′ |  |
| HBdps-f | 5' CGCGGATCCTTCGTGGCCATGGTGCTGTGTCAG GATACGGATTA 3' | To amplify *dps* gene for complementation |
| HBdps-r | 5' CCCAAGCTTTCAGCTCTTTTCCTGCACATGGGCT TCGAGGAACC 3' |  |
| SZbfr-f | 5' CGCGGATCCATGAAGAGAACAGGAGAGCGGACAT 3' | To amplify *bfr* gene for bacterial two hybrid |
| SZbfr-r | 5' CCGGAATTCTTATTCCGCTTCGTTGGCAGAATCG 3' |  |
| SZdps-f | 5' CCGGAATTCATGAAGACGCACAAGACGAAGAACG 3' | To amplify *dps* gene for bacterial two hybrid |
| SZdps-r | 5' CGCGGATCCTCAGCTCTTTTCCTGCACATGGGCT 3' |  |

The underlines indicate restriction sites.
